# Supplementary material for: Modulation of insulin secretion by RBFOX2-mediated alternative splicing
Source: Nat Commun. 2023 Nov 25;14:7732. doi: 10.1038/s41467-023-43605-4 (PMC10676425; doi:10.1038/s41467-023-43605-4)
Supplement: Supplementary file 1 — Supplementary Information [file 41467_2023_43605_MOESM1_ESM.pdf]

Modulation of insulin secretion by RBFOX2-mediated alternative splicing

Supplementary Figure 1

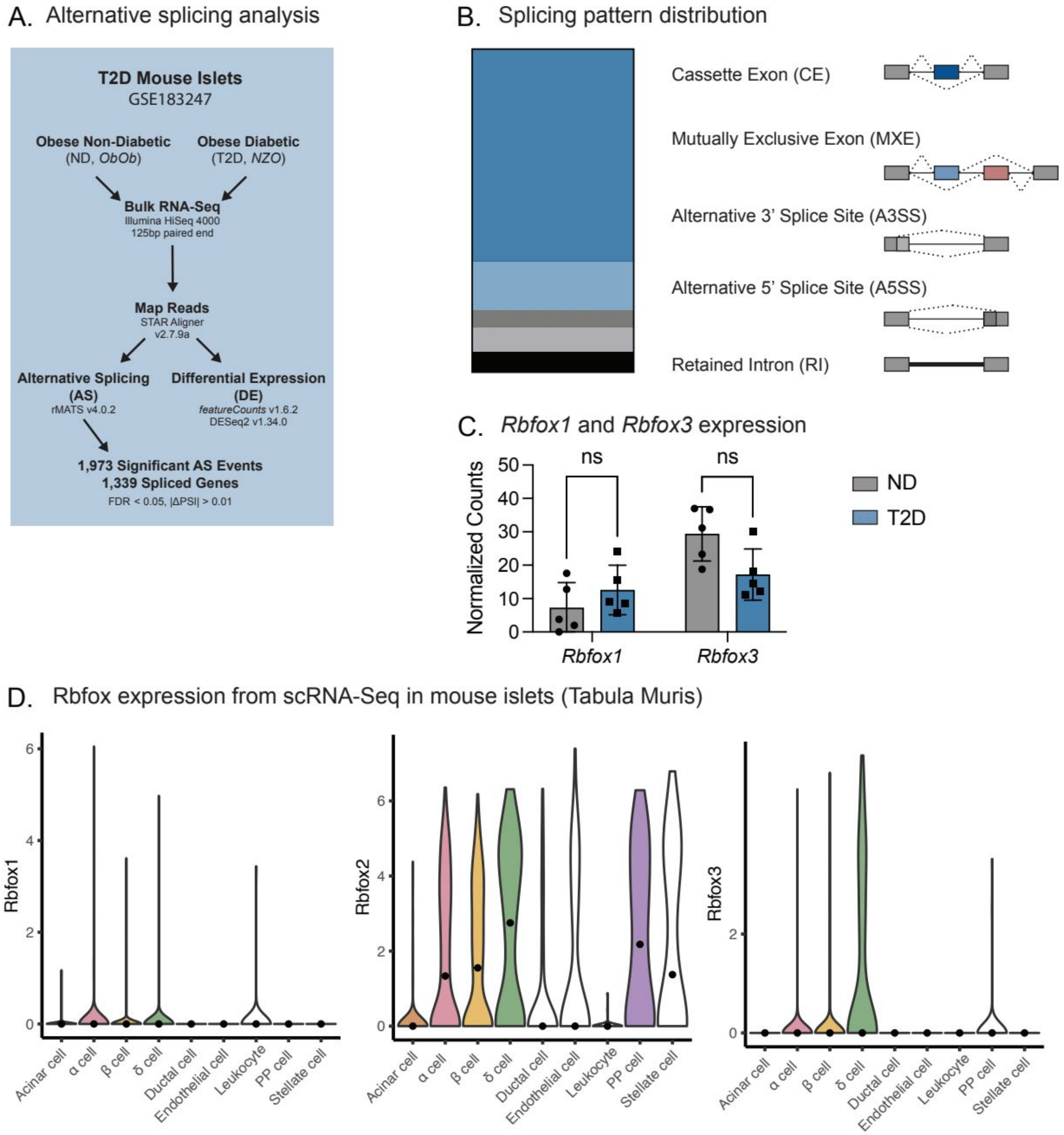

Supplementary Figure 1. Related to Figure 1, RBFOX expression in mouse. (A) Analysis pipeline for comparing alternative splicing and gene expression in obese (ND, *ObOb*) to obese diabetic (T2D, NZO) mouse islets (GSE183247). (B) Splicing pattern distribution of alternative splicing event types (GSE183247). (C) *Rbfox1* ($p = 0.37$) and *Rbfox3* ($p = 0.06$) expression in T2D islets compared to ND controls (GSE183247). (D) *Rbfox1, Rbfox2*, and *Rbfox3* expression in mouse islet cell types (GSE109774).

Supplementary Figure 2

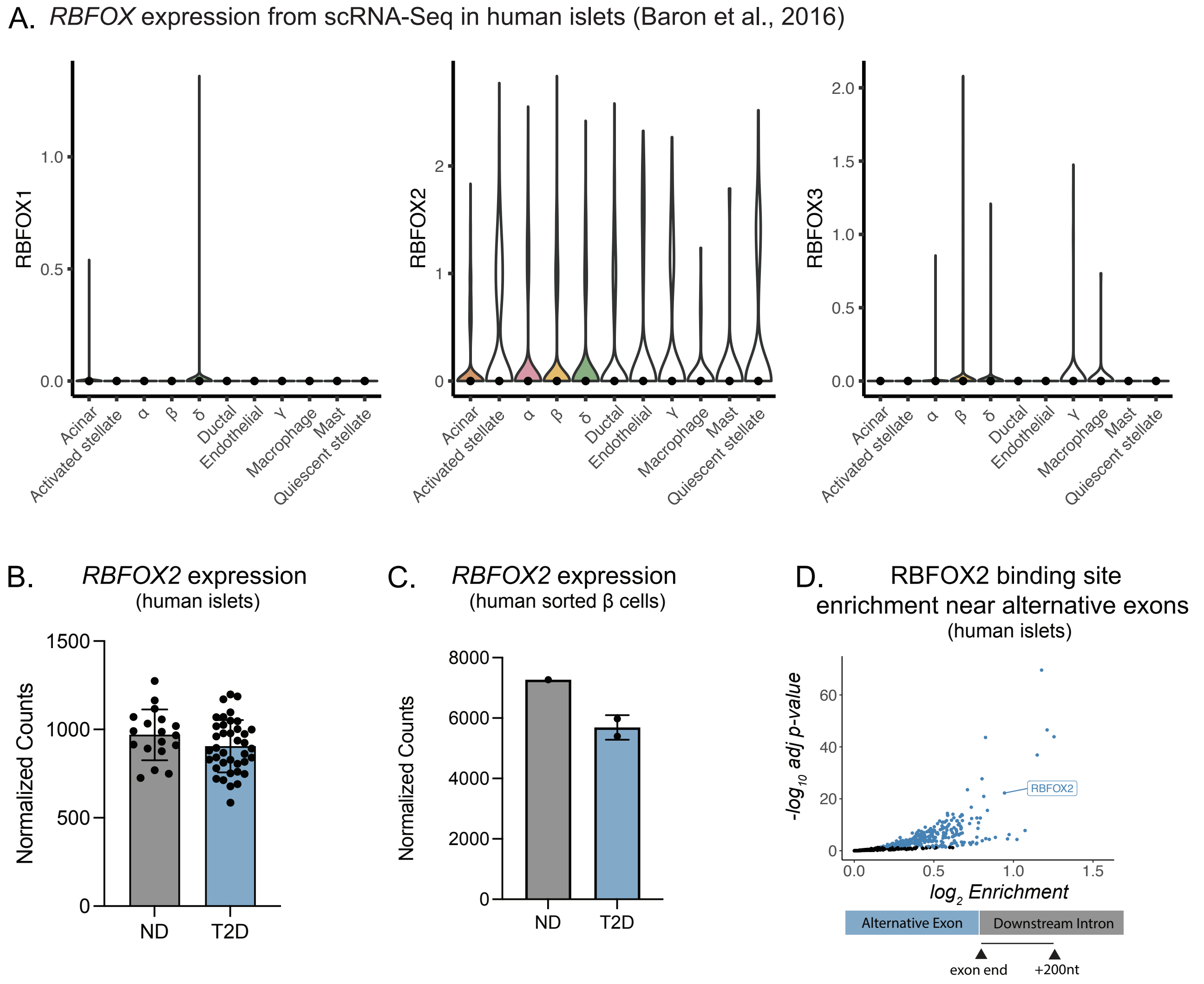

Supplementary Figure 2. Related to Figure 1, *RBFOX2* expression in humans. (A) *Rbfox1, Rbfox2*, and *Rbfox3* expression in human islet cell types (GSE84133). (B) *RBFOX2* expression in human islets from non-diabetic (ND, n = 18) and donors with type 2 diabetes (T2D, n = 39), DESeq2, p = 0.1462 GSE164416). (C) *RBFOX2* expression in sorted human β cells from non-diabetic (ND, n = 1) and donors with type 2 diabetic (T2D, n = 2) (PANC-DB). (D) 5-mer enrichment 200nt downstream of alternative cassette exons identified from rMATS (FDR < 0.05, ΔPSI > 0.01) in non-diabetic (ND, n = 18) and type 2 diabetic (T2D, n = 39) human islets (GSE164416), enrichment relative to constitutive exons plotted adj p-value determined by binomial test and Bonferroni correction. (Data are represented as mean values with SD, ns p > 0.05, * p ≤ 0.05, ** p ≤ 0.01, *** p≤ 0.001, **** p < 0.0001)

Supplementary Figure 3

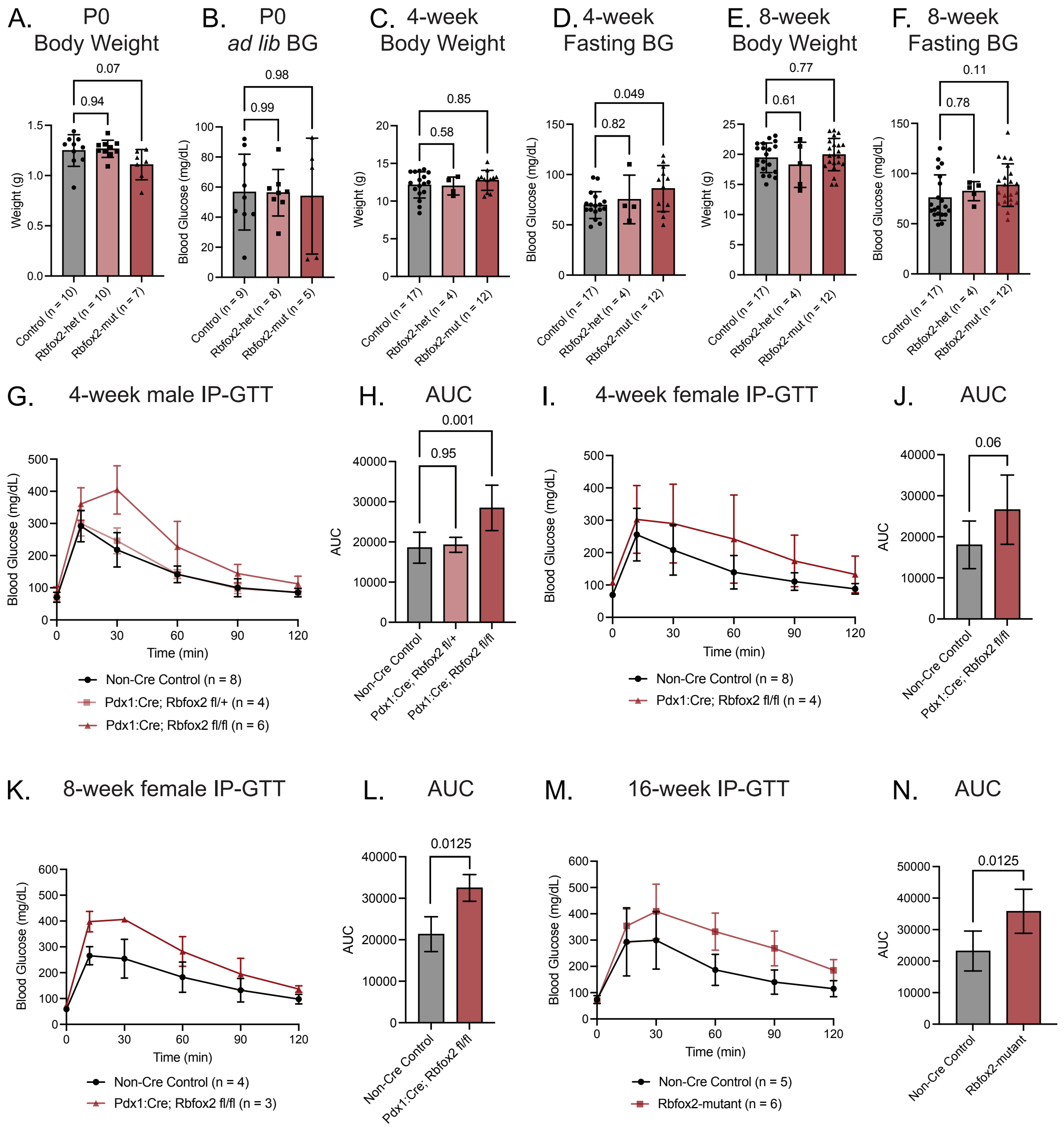

Supplementary Figure 3. Related to Figure 2, *Rbfox2*-mutant mouse characterization. (A) Post-natal day 0 (P0) body weight for control (Rbfox2$^{fl/fl}$, n = 9), *Rbfox2*-het (Pdx1:CRE; Rbfox2$^{fl/+}$, n = 8), and *Rbfox2*-mut (Pdx1:CRE; Rbfox2$^{fl/fl}$, n = 8), one-way ANOVA with multiple comparisons, F = 2.289. (B) Post-natal day 0 (P0) *ad lib* blood glucose (BG) for control (n = 9), *Rbfox2*-het (Pdx1:CRE; n = 8), and *Rbfox2*-mut (n = 8), one-way ANOVA with multiple comparisons, F = 0.018. (C) 4-week body weight for control (n = 17), *Rbfox2*-het (n = 4), and *Rbfox2*-mut (n = 12), one-way ANOVA with multiple comparisons, F = 0.5698. (D) 4-week fasting blood glucose (BG) for control (n = 17), *Rbfox2*-het (n = 4), and *Rbfox2*-mut (n = 12), one-way ANOVA with multiple comparisons, F = 2.764. (E) 8-week body weight for control (n = 17), *Rbfox2*-het (n = 4), and *Rbfox2*-mut (n = 12), one-way ANOVA with multiple comparisons, F = 0.8367. (F) 8-week fasting blood glucose BG) for control (n = 17), *Rbfox2*-het (n = 4), and *Rbfox2*-mut (n = 12), one-way ANOVA with multiple comparisons, F = 1.857. (G) Intraperitoneal glucose tolerance test (IP-GTT) in 4-week male mice, control (*Rbfox2*$^{fl/fl}$, n = 8), *Rbfox2-het* (*Pdx1*:CRE; *Rbfox2*$^{fl/+}$, n = 4) and *Rbfox2-mut* (*Pdx1*:CRE; *Rbfox2*$^{fl/fl}$, n = 6). (H) Area under the curve (AUC) for 4-week male IP-GTT, one-way ANOVA with multiple comparisons F = 10.23. (I) IP-GTT in 4-week female mice, control (n = 8) and *Rbfox2-mut* (n = 4). (J) Area under the curve (AUC) for 4-week female IP-GTT, two-tailed t-test. (K) IP-GTT in 8-week female mice, control (n = 4) and *Rbfox2-mut* (n = 3). (L) Area under the curve (AUC) for 8-week female IP-GTT, two-tailed t-test. (M) IP-GTT in 16-week combined male and female mice, control (n = 4) and *Rbfox2-mut* (n = 3). (N) Area under the curve (AUC) for 16-week combined male and female IP-GTT, two-tailed t-test. (Data are represented as mean values with SD, ns $p > 0.05$, * $p \leq 0.05$, ** $p \leq 0.01$, *** $p \leq 0.001$, **** $p < 0.0001$)

Supplementary Figure 4

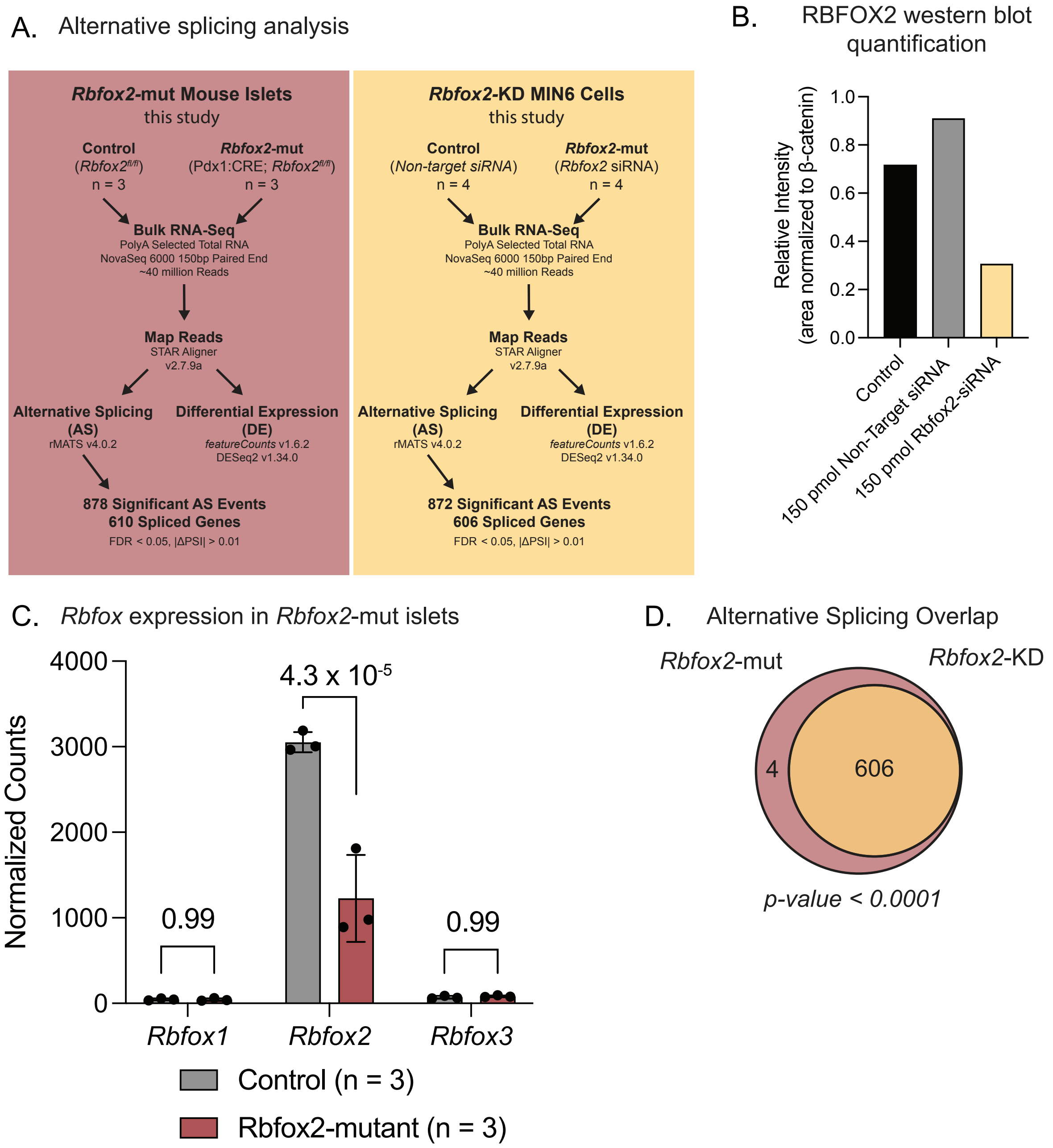

Supplementary Figure 4. Related to Figure 3, Comparison of *Rbfox2*-mut mouse model and *Rbfox2*-KD MIN6 cell model. (A) Analysis pipeline for comparing alternative splicing and gene expression both *Rbfox2*-mut islet bulk-RNA-Seq and *Rbfox2*-KD MIN6 cell bulk-RNA-Seq. (B) Quantification of RBFOX2 western from 150pmol *Rbfox2*-siRNA treatment in MIN6 cells. (C) *Rbfox1, Rbfox2,* and, *Rbfox3* expression in *Rbfox2*-mut islets, significance determined by DESeq2. (D) Overlap of alternatively spliced gene in *Rbfox2*-mut islets and *Rbfox2*-KD MIN6 cells at 1% ΔPSI, significant overlap determined by hypergeometric analysis.

Supplementary Figure 5

A. Alternative Splicing Overlap

Overlap of alternatively spliced genes in experimental models

ΔPSI	Overlap	Islet Specific	MIN6 Specific
1%	606	4	0
5%	427	4	0
10%	230	4	0
25%	41	0	0

p-value < 0.005

B. Splicing pattern distribution

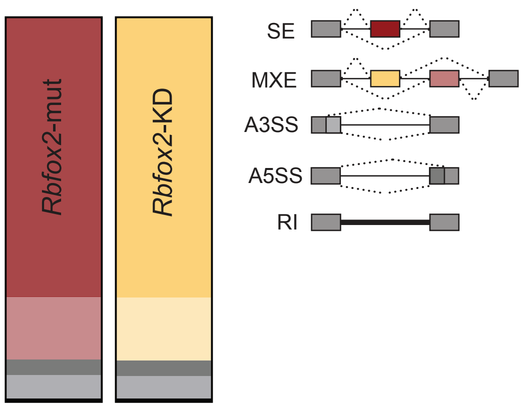

C. Comparison of ΔPSI by splicing event *Rbfox2*-KD MIN6 and *Rbfox2*-mut Islet

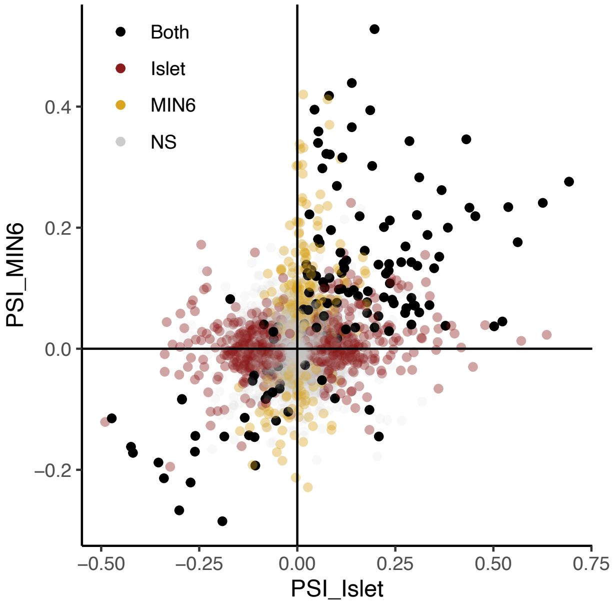

D. Comparison of ΔPSI by splicing event T2D and *Rbfox2*-mut Islet

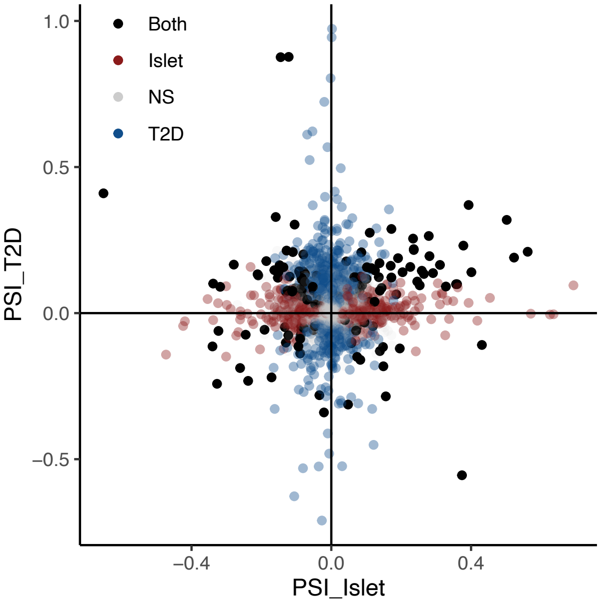

E. Overlapping alternatively spliced genes (5% ΔPSI)

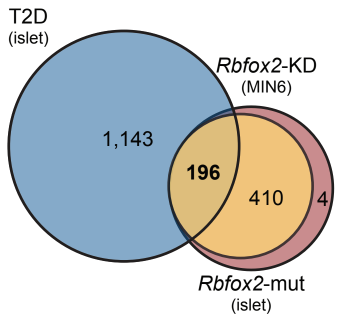

F. GO Terms for overlapping genes (5% ΔPSI)

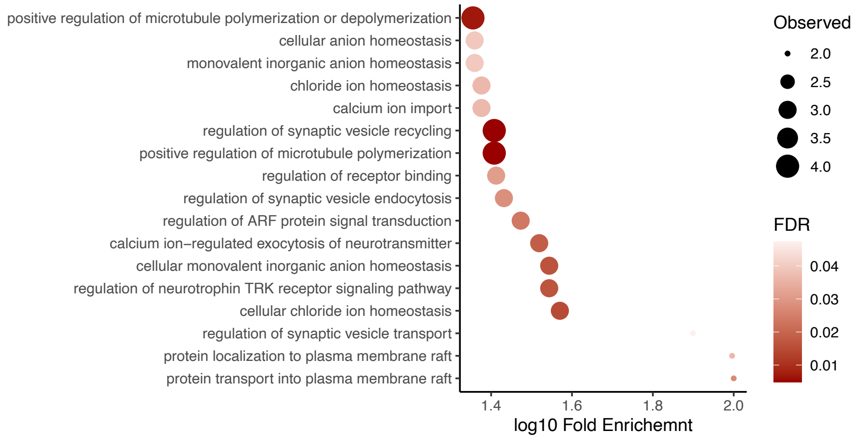

Supplementary Figure 5. Related to Figure 3, Gene and exon level splicing overlap of *Rbfox2*-KD and *Rbfox2*-mut at 5% ΔPSI. (A) Overlap of alternatively spliced genes between *Rbfox2*-KD MIN6 cells and *Rbfox2*-mut islets with increasing ΔPSI thresholds. (B) Splicing event type distribution for *Rbfox2*-KD MIN6 and *Rbfox2*-mut islet as identified by rMATS, 1% ΔPSI cutoff. (C) Correlation between *Rbfox2*-KD MIN6 and *Rbfox2*-mut islet splicing events at the level of the exon, point color indicates significance – significant in both datasets (black), significant in *Rbfox2*-KD only (yellow), significant in *Rbfox2*-mut only (red), not significant (gray). (D) Correlation between *Rbfox2*-mut islet and T2D islet splicing events at the level of the exon, point color indicates significance – significant in both datasets (black), significant in *Rbfox2*-mut only (red), significant in T2D only (blue), not significant (gray). (E) Overlap of conserved alternative splicing events at 5% ΔPSI across *Rbfox2*-KD MIN6, *Rbfox2*-mut islets, and T2D mouse islets (GSE183247). (F) GO Terms for 112 significantly alternatively spliced (ΔPSI > 0.05) genes shared across *Rbfox2*-KD MIN6, *Rbfox2*-mut islets, and T2D mouse islets (GSE183247).

Supplementary Figure 6

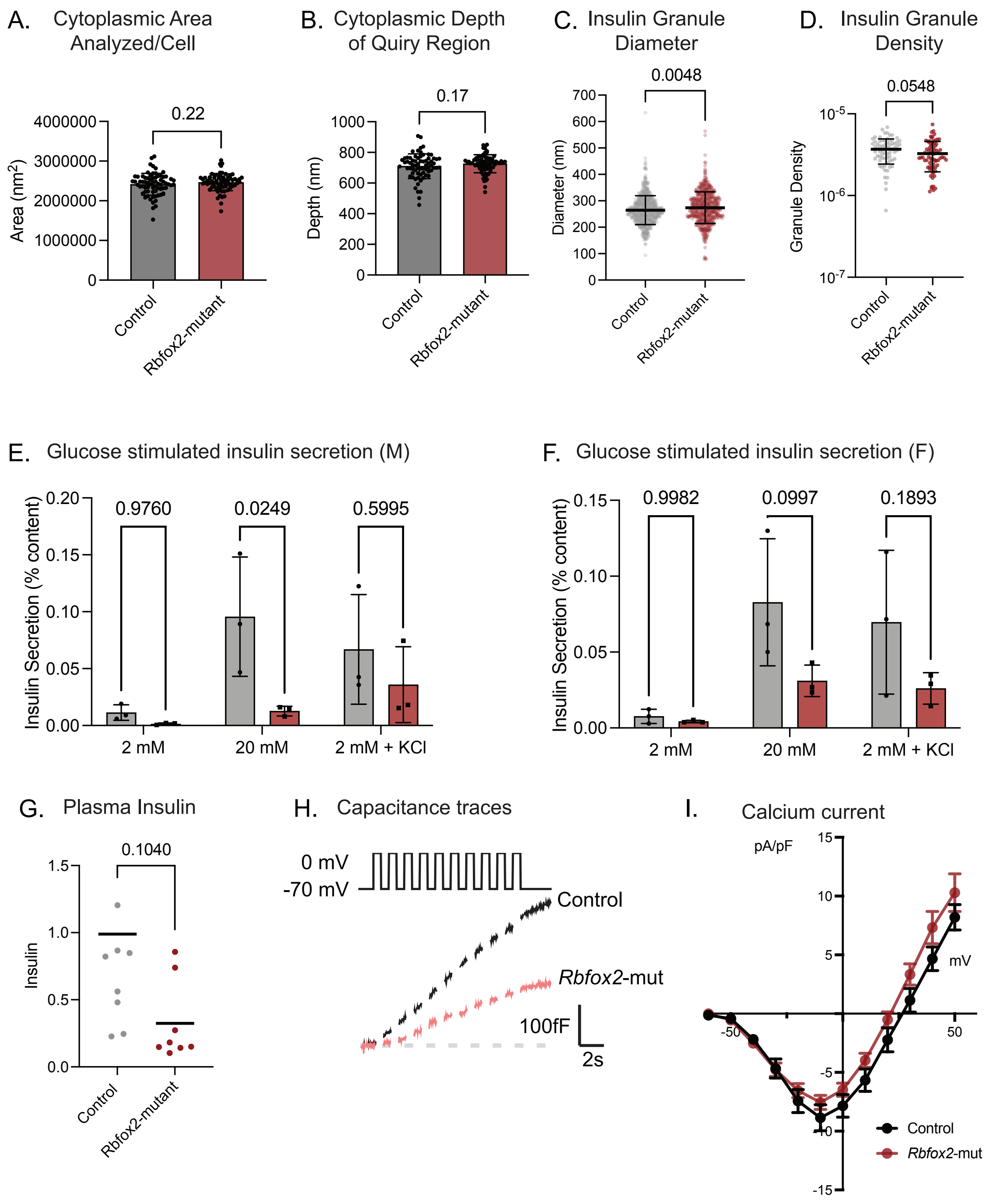

Supplementary Figure 6. Related to Figure 4, Insulin secretion and insulin granule quantification. (A) Cytoplasmic area quantified in insulin granule analysis, each dot is a cell, two-tailed t-test. (B) Cytoplasmic depth quantified in insulin granule analysis, each dot represents a cell, two-tailed t-test. (C) Insulin granule diameter, each dot represents an insulin granule, t-test. (D) Insulin granule density, each dot represents a β cell quantified, two-tailed t-test. (E) Glucose stimulated insulin secretion measured by ELISA, 8-week male mice, n = 3 mice per group, two-way ANOVA with multiple comparisons. (F) Glucose stimulated insulin secretion measured by ELISA, 8-week female, n = 3 mice per group, two-way ANOVA with multiple comparisons. (G) Plasma insulin measured by ELISA 15 min post glucose injection, n = 8 control and n = 7 mutant mice, t-test containing outlier, two-tailed t-test. (H) Capacitance trace. (I) Calcium current measurement for *Rbfox2*-mut and control β cells. (Data are represented as mean values with SD , ns $p > 0.05$, * $p \leq 0.05$, ** $p \leq 0.01$, *** $p \leq 0.001$, **** $p < 0.0001$)

Supplementary Figure 7

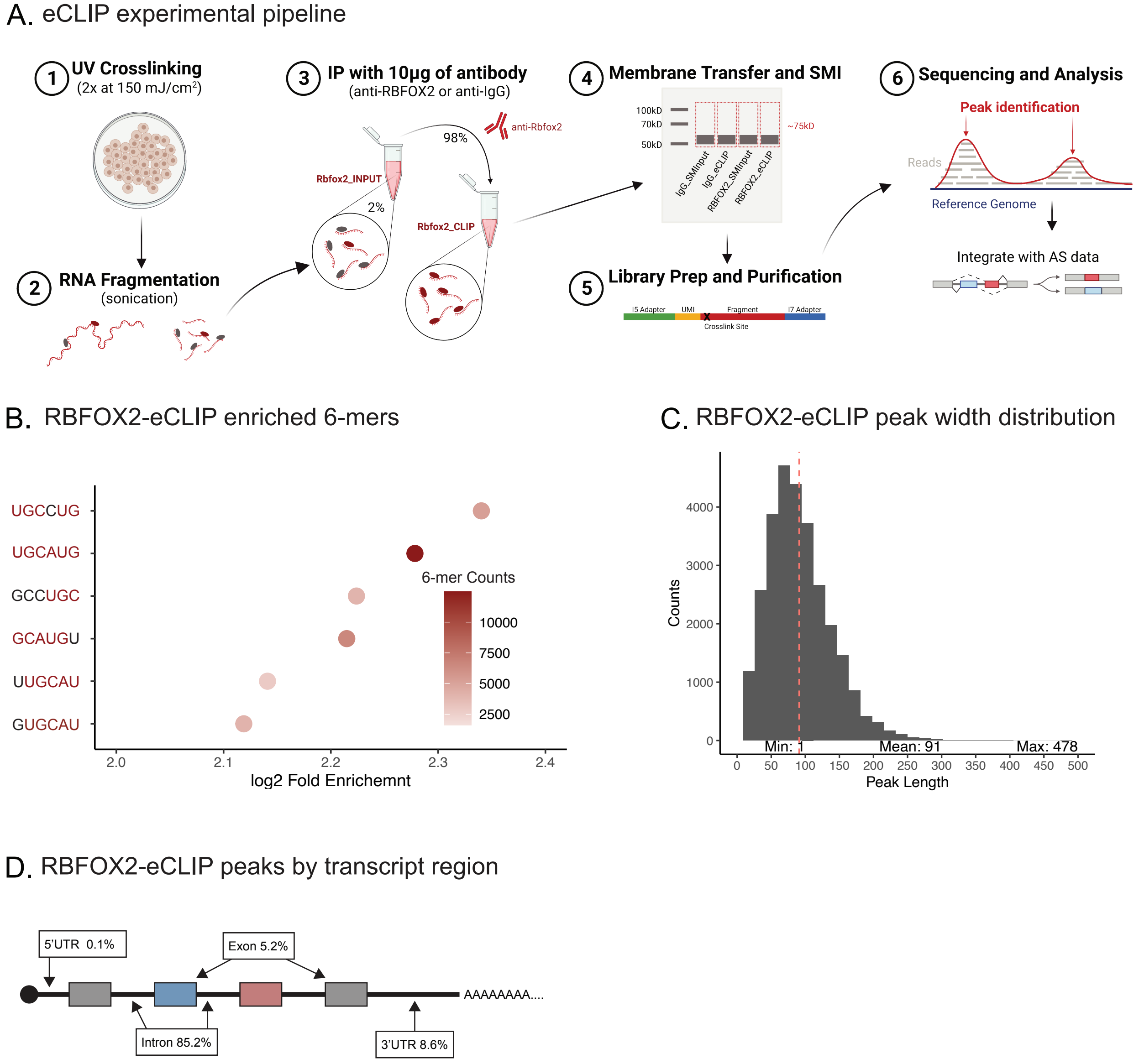

Supplementary Figure 7. Related to Figure 5, RBFOX2-eCLIP sequencing and analysis. (A) RBFOX2-eCLIP sequencing experimental pipeline – 1. UV crosslinking twice at 150 mJ/cm^2, 2. RNA fragmentation by sonication, 3. IP with RBFOX2 and IgG antibody, 4. Size-matched input (SMI) collection and reverse crosslinking, 5. Library preparation, 6. Sequencing and analysis (BioRender). (B) Enriched 6-mers under significant RBFOX2 eCLIP peaks ($p < 0.05$, $log_2FC > 0$). (C) RBFOX2-eCLIP peak width distribution ($p < 0.05$, $log_2FC > 0$). (D) RBFOX2-eCLIP peak distribution within genes ($p < 0.05$, $log_2FC > 0$).

Supplementary Figure 8

A. RBFOX2-eCLIP enriched 6-mers (Rep1)

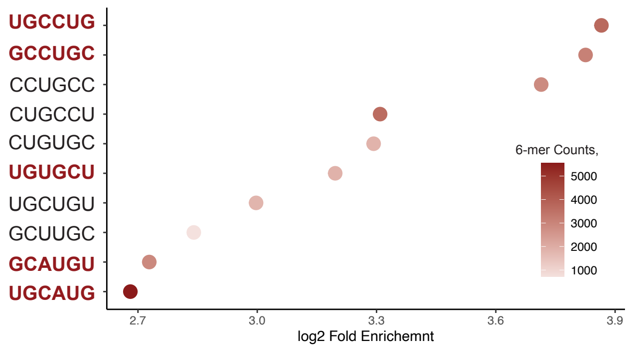

B. RBFOX2-eCLIP enriched 6-mers (Rep2)

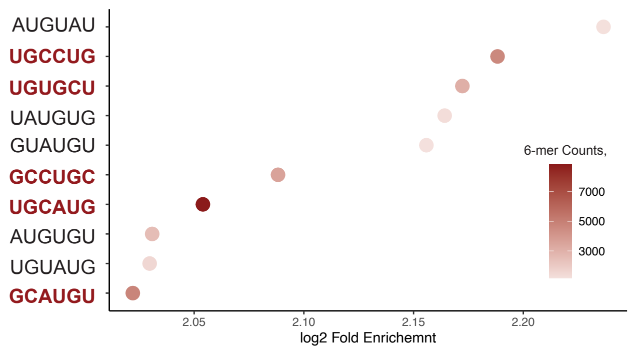

C. RBFOX2-eCLIP peak width distribution (Rep1)

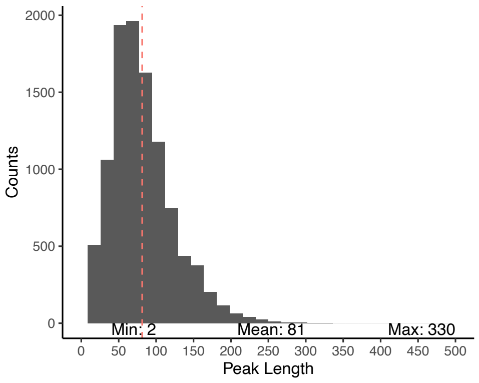

D. RBFOX2-eCLIP peak width distribution (Rep2)

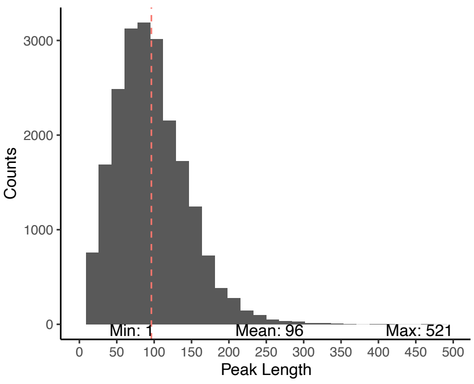

E. RBFOX2-eCLIP peaks by transcript region (Rep1)

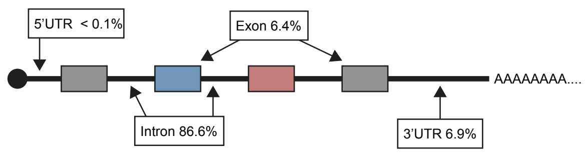

F. RBFOX2-eCLIP peaks by transcript region (Rep2)

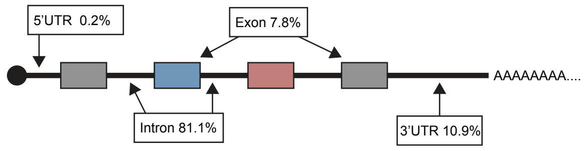

G. RBFOX2-eCLIP replicate comparison

	RBFOX2-eCLIP	Rep1	Rep2
**Clipper output filtered for p < 0.05 and log_2FC > 0.**	30993	10395	21285
Peaks in genes, log_2FC > 0, p < 0.05, and remove "Gm" and "Rik"	20603	7596	13982
Peaks in genes, log_2FC > 0, p $<10^{-3}$	6537	2397	4151
**Peaks in gene, Fold Enrichment ≥ 2, p $< 10^{-3}$**	6514	2387	4138
3'UTR	559	165	450
5'UTR	8	1	10
Intron	5548	2068	3356
Exon	339	153	322
**Genes with peaks**	2494	1238	1774
**Alternatively spliced genes**	606	606	606
**Genes with peaks and AS**	251	140	206
How many splicing events are in RBFOX2 bound genes?	411	234	342
**SE**	304	174	262
Inclusion	224	124	189
Skipping	80	50	73
**MXE**	78	43	60
MXE Inclusion (1st Exon Included)	31	21	23
MXE Skipping (1st Exon Skipped)	47	22	37
**A3SS**	18	10	11
**A5SS**	10	7	8
**RI**	1	0	1
**Self-consistency ratio**		1.2	
**Rescue ratio**		2.3	
**IDR 0.01 replicating peaks**		3300	

Supplementary Figure 8. Related to Figure 5, RBFOX2-eCLIP replicate analysis. (A) Enriched 6-mers under significant RBFOX2 eCLIP peaks ($p < 0.05$, $log_2FC > 0$) for replicate 1. (B) Enriched 6-mers under significant RBFOX2 eCLIP peaks ($p < 0.05$, $log_2FC > 0$) for replicate 2. (C) RBFOX2-eCLIP peak width distribution ($p < 0.05$, $log_2FC > 0$) for replicate 1. (D) RBFOX2-eCLIP peak width distribution ($p < 0.05$, $log_2FC > 0$) for replicate 2. (E) RBFOX2-eCLIP peak distribution within genes replicate 1 ($p < 0.05$, $log_2FC > 0$). (F) RBFOX2-eCLIP peak distribution within genes replicate 2 ($p < 0.05$, $log_2FC > 0$). G. RBFOX2-eCLIP replicate comparison summary.

Supplementary Figure 9

A. RBFOX2 bound and alternatively spliced genes

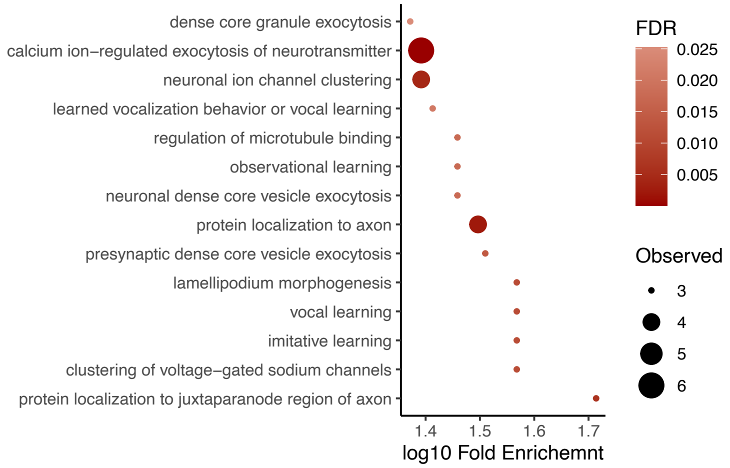

B. *Syt7* alternative splicing and RBFOX2 binding

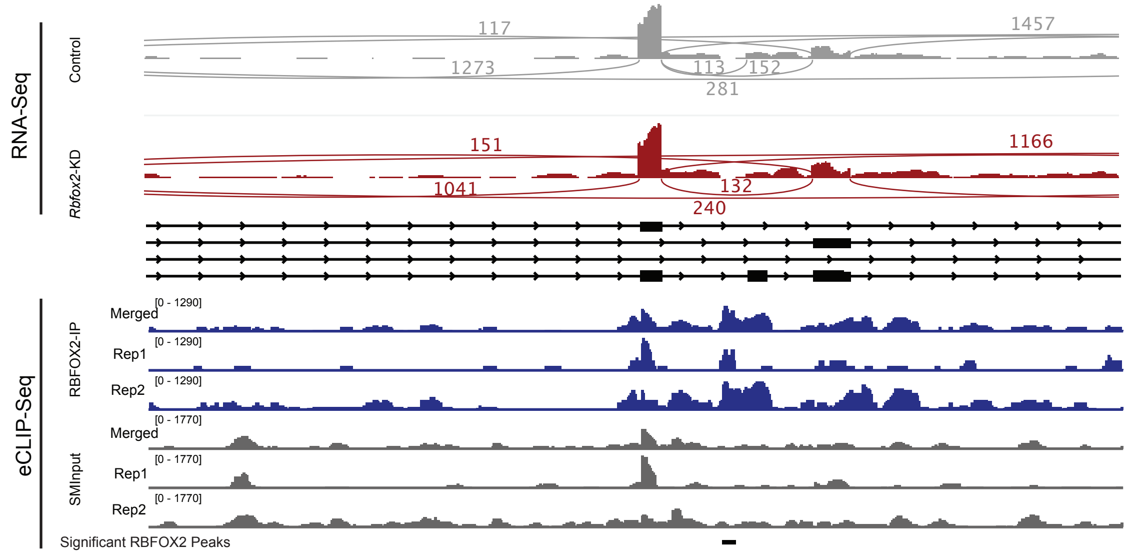

C. *Stxbp1* alternative splicing and RBFOX2 binding

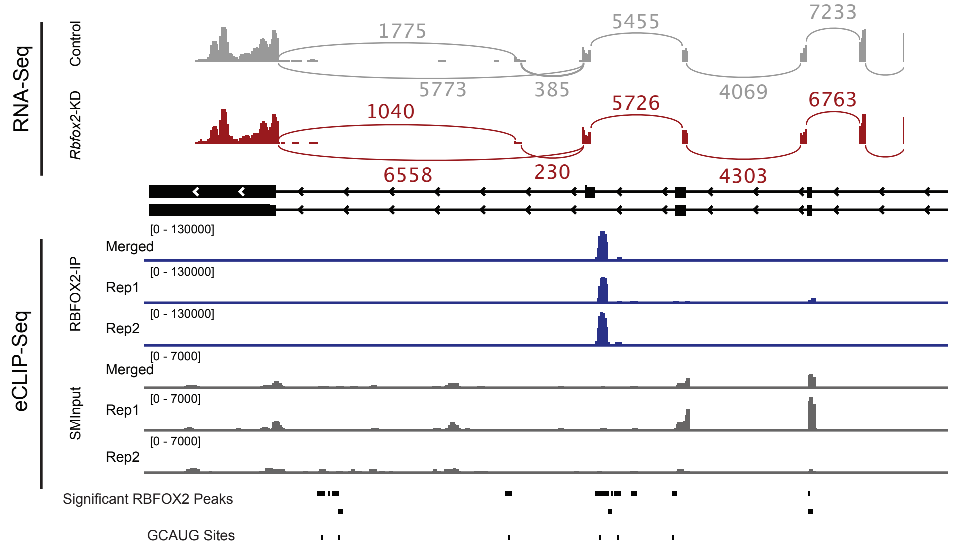

Supplementary Figure 9. Related to Figure 6, RBFOX2 regulation of additional SNARE complex components. (A) GOTerms for RBFOX2 bound and alternatively spliced transcripts. (B) Sashimi plot for *Syt7* SE with eCLIP peaks, RBFOX2 eCLIP-Seq (blue), *clipper* identified significant peaks (black bars), SMI eCLIP-Seq (gray). *Syt7* ΔPSI = 0.06, FDR = 0.017. (C) Sashimi plot for *Stxbp1* SE with eCLIP peaks, RBFOX2 eCLIP-Seq (blue), *clipper* identified significant peaks (black bars), GCAUG sequence (black bars), SMI eCLIP-Seq (gray). *Stxbp1* ΔPSI = 0.071, FDR < 0.0001.
