# Supplementary material for: Modulation of insulin secretion by RBFOX2-mediated alternative splicing
Source: Nat Commun. 2023 Nov 25;14:7732. doi: 10.1038/s41467-023-43605-4 (PMC10676425; doi:10.1038/s41467-023-43605-4)
Supplement: Supplementary file 3 — Description of Additional Supplementary Files [file 41467_2023_43605_MOESM3_ESM.pdf]

<u>**Description of Additional Supplementary Files**</u>

Supplementary Data 1. Related to Figure 1 and Figure S1. Significant alternative splicing events comparing obese (ND, Ob/Ob, n = 5) to obese diabetic (T2D, NZO, n = 5) mouse islets (GSE183247). Statistical analysis was conducted by rMATs and filtered for FDR < 0.05 and _PSI > 0.01.

Supplementary Data 2. Related to Figure 1 and Figure S1. rMATS results identifying cassette or skipped exon (SE) splicing events comparing obese (ND, Ob/Ob, n = 5) to obese diabetic (T2D, NZO, n = 5) mouse islets (GSE183247).

Supplementary Data 3. Related to Figure 1 and Figure S1. rMATS results identifying mutually exclusive exon (MXE) splicing events comparing obese (ND, Ob/Ob, n = 5) to obese diabetic (T2D, NZO, n = 5) mouse islets (GSE183247).

Supplementary Data 4. Related to Figure 1 and Figure S1. rMATS results identifying alternative 3' splice site (A3SS) splicing events comparing obese (ND, ObOb, n = 5) to obese diabetic (T2D, NZO, n = 5) mouse islets (GSE183247).

Supplementary Data 5. Related to Figure 1 and Figure S1. rMATS results identifying alternative 5' splice site (A5SS) splicing events comparing obese (ND, ObOb, n = 5) to obese diabetic (T2D, NZO, n = 5) mouse islets (GSE183247).

Supplementary Data 6. Related to Figure 1 and Figure S1. rMATS results identifying retained intron (RI) splicing events comparing obese (ND, ObOb, n = 5) to obese diabetic (T2D, NZO, n = 5) mouse islets (GSE183247).

Supplementary Data 7. Related to Figure 1 and Figure S1. DESeq2 results identifying differential expression of RNA binding proteins (GO:0003729) comparing obese (ND, ObOb, n = 5) to obese diabetic (T2D, NZO, n = 5) mouse islets (GSE183247).

Supplementary Data 8. Related to Figure 1. 5-mer enrichment 200nt downstream of alternative cassette/skipped exons identified from rMATS (FDR < 0.05, ΔPSI > 0.01) in ND (ob/ob, n = 5) and T2D (NZO, n = 5) mouse islets (GSE183247), enrichment relative to constitutive exons plotted adj p-value from binomial test with Bonferroni correction.

Supplementary Data 9. Related to Figure S4. rMATS results identifying cassette or skipped exon (SE) splicing events comparing control (Rbfox2fl/fl, n = 3) and Rbfox2-mut (Pdx1:CRE; Rbfox2fl/fl, n = 3) islets.

Supplementary Data 10. Related to Figure S4. rMATS results identifying cassette or mutually exclusive exon (MXE) splicing events comparing control (Rbfox2fl/fl, n = 3) and Rbfox2-mut (Pdx1:CRE; Rbfox2fl/fl, n = 3) islets.

Supplementary Data 11. Related to Figure S4. rMATS results identifying cassette or alternative 3' splice site (A3SS) splicing events comparing control (Rbfox2fl/fl, n = 3) and Rbfox2-mut (Pdx1:CRE; Rbfox2fl/fl, n = 3) islets.

Supplementary Data 12. Related to Figure S4. rMATS results identifying cassette or alternative 5' splice site (A5SS) splicing events comparing control (Rbfox2fl/fl, n = 3) and Rbfox2-mut (Pdx1:CRE; Rbfox2fl/fl, n = 3) islets.

Supplementary Data 13. Related to Figure S4. rMATS results identifying cassette or retained intron (RI) splicing events comparing control (Rbfox2fl/fl, n = 3) and Rbfox2-mut (Pdx1:CRE; Rbfox2fl/fl, n = 3) islets.

Supplementary Data 14. Related to Figure 3 and Figure S5. Cassette/skipped exon alternative splicing overlap at the exon level across the T2D islets (GSE183247), Rbfox2-KD in MIN6 cells (this study), and Rbfox2-mut islets. Statistical analysis was conducted by rMATs and filtered for FDR < 0.05 and ΔPSI > 0.01.

Supplementary Data 15. Related to Figure 5 and Figure S7. Significant RBFOX2-eCLIP peaks in MIN6 cells (n = 2 experimental replicates, statistical analysis conducted by Clipper additional details are available DOI: 10.5281/zenodo.8338787 and DOI:10.5281/zenodo.8335301).

Supplementary Data 16. Related to Figure 5B-E and Figure S7. Normalized RBFOX2-eCLIP peak counts near alternative exons.

Supplementary Data 17. Related to Figure 6. PSI of conserved significant RBFOX2 bound cassette exon splicing events across Rbfox2-KD in MIN6, Rbfox2-mut mouse islets, and T2D mouse islets (GSE183247).

Supplementary Data 18. Probes and Primers

Supplementary Data 19. Resources and Reagents

Supplementary Data 20. Published data analysis pipelines used in the study.
