# Supplementary material for: Modulation of insulin secretion by RBFOX2-mediated alternative splicing
Source: Nat Commun. 2023 Nov 25;14:7732. doi: 10.1038/s41467-023-43605-4 (PMC10676425; doi:10.1038/s41467-023-43605-4)
Supplement: Supplementary file 24 — Reporting Summary [file 41467_2023_43605_MOESM24_ESM.pdf]

nature portfolio

Corresponding author(s): Lori Sussel

Last updated by author(s): Sep 25, 2023

Reporting Summary

Nature Portfolio wishes to improve the reproducibility of the work that we publish. This form provides structure for consistency and transparency in reporting. For further information on Nature Portfolio policies, see our Editorial Policies and the Editorial Policy Checklist.

Statistics

For all statistical analyses, confirm that the following items are present in the figure legend, table legend, main text, or Methods section.

n/a	Confirmed	
☐	☒	The exact sample size (*n*) for each experimental group/condition, given as a discrete number and unit of measurement
☐	☒	A statement on whether measurements were taken from distinct samples or whether the same sample was measured repeatedly
☐	☒	The statistical test(s) used AND whether they are one- or two-sided<br>*Only common tests should be described solely by name; describe more complex techniques in the Methods section.*
☐	☒	A description of all covariates tested
☐	☒	A description of any assumptions or corrections, such as tests of normality and adjustment for multiple comparisons
☐	☒	A full description of the statistical parameters including central tendency (e.g. means) or other basic estimates (e.g. regression coefficient) AND variation (e.g. standard deviation) or associated estimates of uncertainty (e.g. confidence intervals)
☐	☒	For null hypothesis testing, the test statistic (e.g. *F*, *t*, *r*) with confidence intervals, effect sizes, degrees of freedom and *P* value noted<br>*Give P values as exact values whenever suitable.*
☒	☐	For Bayesian analysis, information on the choice of priors and Markov chain Monte Carlo settings
☒	☐	For hierarchical and complex designs, identification of the appropriate level for tests and full reporting of outcomes
☒	☐	Estimates of effect sizes (e.g. Cohen's *d*, Pearson's *r*), indicating how they were calculated

Our web collection on statistics for biologists contains articles on many of the points above.

Software and code

Policy information about availability of computer code

Data collection	Data is documented in Original code and pipelines are deposited at https://github.com/CUAnschutzBDC and are publicly available
Data analysis	Original code and pipelines are deposited at https://github.com/CUAnschutzBDC and are publicly available

For manuscripts utilizing custom algorithms or software that are central to the research but not yet described in published literature, software must be made available to editors and reviewers. We strongly encourage code deposition in a community repository (e.g. GitHub). See the Nature Portfolio guidelines for submitting code & software for further information.

Data

Policy information about availability of data

All manuscripts must include a data availability statement. This statement should provide the following information, where applicable:

- Accession codes, unique identifiers, or web links for publicly available datasets
- A description of any restrictions on data availability
- For clinical datasets or third party data, please ensure that the statement adheres to our policy

This paper re-analyzes existing publicly available data located at GEO - GSE183247 [https://www.ncbi.nlm.nih.gov/geo/query/acc.cgi?acc=GSE183247] originally published by Schurmann et al. 21; GSE164416 [https://www.ncbi.nlm.nih.gov/geo/query/acc.cgi?acc=GSE164416] originally published by Wigger et al. 40; and PANC-DB [https://hpap.pmacs.upenn.edu/explore/download?matrix] originally published by Kaestner et al. 41. The accession numbers for the data generated in this study are available through the SuperSeries accession number GSE221277 [https://www.ncbi.nlm.nih.gov/geo/query/acc.cgi?acc=GSE221277], or individually at

GSE221274 [https://www.ncbi.nlm.nih.gov/geo/query/acc.cgi?acc=GSE221274] (Rbfox2-KD RNA-Seq MIN6), GSE221275 [https://www.ncbi.nlm.nih.gov/geo/query/acc.cgi?acc=GSE221275] (Rbfox2-mut RNA-Seq Mouse Islet), and GSE221276 [https://www.ncbi.nlm.nih.gov/geo/query/acc.cgi?acc=GSE221276] (RBFOX2 eCLIP-Seq MIN6). The URL links for all software tools are listed in Supplementary Table 20. Source data are provided as a Source Data File.

Research involving human participants, their data, or biological material

Policy information about studies with human participants or human data. See also policy information about sex, gender (identity/presentation), and sexual orientation and race, ethnicity and racism.

Reporting on sex and gender	N/A
Reporting on race, ethnicity, or other socially relevant groupings	N/A
Population characteristics	N/A
Recruitment	N/A
Ethics oversight	N/A

Note that full information on the approval of the study protocol must also be provided in the manuscript.

Field-specific reporting

Please select the one below that is the best fit for your research. If you are not sure, read the appropriate sections before making your selection.

☒ Life sciences ☐ Behavioural & social sciences ☐ Ecological, evolutionary & environmental sciences

For a reference copy of the document with all sections, see nature.com/documents/nr-reporting-summary-flat.pdf

Life sciences study design

All studies must disclose on these points even when the disclosure is negative.

Sample size	A minimum sample size of 3 for control and experimental groups were used in each assay, these numbers were determined by previous experiments in our lab and in the field. Replicates for GTT analysis were 4-8 Based on previous experiments in the lab these numbers will give 90% power to detect a treatment effect size of 65-75% at α = 0.05 for a Two-Way ANOVA.
Data exclusions	A single outlier was identified and removed from the plasma insulin measurements using the exclusion criteria ROUT (Q = 0.5%) method.
Replication	A minimum of 3-4 biological replicates for both mouse studies and cell line assays were conducted. Technical replicates for each assay. All experimental findings were confirmed by replication.
Randomization	When possible, samples were randomly separated into groups. In genetic mouse model experiments, littermate controls were selected for comparison when possible.
Blinding	Double-blinded methods were used when applicable, specifically during EM sample prep and image quantification.

Reporting for specific materials, systems and methods

We require information from authors about some types of materials, experimental systems and methods used in many studies. Here, indicate whether each material, system or method listed is relevant to your study. If you are not sure if a list item applies to your research, read the appropriate section before selecting a response.

Materials & experimental systems

n/a	Involved in the study	
☐	☒	Antibodies
☐	☒	Eukaryotic cell lines
☒	☐	Palaeontology and archaeology
☐	☒	Animals and other organisms
☒	☐	Clinical data
☒	☐	Dual use research of concern
☒	☐	Plants

Methods

n/a	Involved in the study	
☒	☐	ChIP-seq
☒	☐	Flow cytometry
☒	☐	MRI-based neuroimaging

Antibodies

Antibodies used	Rabbit anti-RBFOX2 (1:5000 for western, 10µg for eCLIP) Bethyl A300-864A<br>Rabbit anti-β-catenin (1:2000) Abcam Ab32572<br>Rabbit anti-HRP (1:5000) Abcam Ab205718<br>Guinea pig anti-INS (1:10) Dako/Agilent Cat# IR00261-2<br>Rat anti-SST (1:500) Abcam Ab30788<br>Rabbit anti-Glucagon (1:250) Cell Signaling Technologies Cat# 2760S RRID:AB_659831<br>Rabbit anti-c-peptide 1 (1:1000) BCBC Ab1042 and 1044<br>488 goat anti-guinea pig (1:500) Thermo Fisher/Invitrogen Cat# A11073 RRID:AB_2534117<br>594 donkey ant-rabbit (1:500) Thermo Fisher/Invitrogen Cat# A21207 RRID:AB_141637<br>488 donkey anti-rat (1:500) Invitrogen A21208<br>555 goat anti-guinea pig (1:500) Thermo Fisher/Invitrogen Cat# A21435 RRID:AB_15006<br>488 donkey anti-rabbit (1:500) Thermo Fisher/Invitrogen Cat# A21206 RRID:AB_141708<br>DAPI (4',6-diamidino-2-phenylindole) (1:1000) Thermo Fisher Cat# D1306 RRID:AB_2629482
Validation	Rabbit anti-RBFOX2 (RBM9) - validated by Bethyl using their 6 Pillar formula (PMID: 27595404). Also validated by authors using western analysis on mouse KD MIN6 cells. Validated for eCLIP in Lorenz et al., Nature Methods 2023 (PMID: 36550273).<br><br>Rabbit anti-β-catenin: validated by Abcam using IHC and western on wild type and knockout tissue.<br><br>Guinea pig anti-INS: Validated by supplier for 100% specificity using an radioimmunoassay. Validated by authors using immunofluorescence on islet tissue from wild type and Nkx2.2 KO mice that lack insulin.<br><br>Rat anti-Glucagon: Validated by supplier for absence of cross reactivity with other preproglucagon peptides. Also validated by authors using immunofluorescence on islet tissue from wild type mice to validate that signal was in cells around the periphery of the islet and did not co-express with other islet hormones or label exocrine tissue.<br><br>Rabbit anti-somatostatin: Validated by authors using immunofluorescence on islet tissue from wild type mice to validate that signal was in cells around the periphery of the islet and did not co-express with other islet hormones or label exocrine tissue.<br><br>Rabbit anti c-peptide: Validated by supplier and members of the NIH Beta Cell Biology Consortium for specificity. Validated by authors using immunofluorescence on islet tissue from wild type mice to validate that signal was in cells co-expressing insulin.

Eukaryotic cell lines

Policy information about cell lines and Sex and Gender in Research

Cell line source(s)	MIN6 cells. The MIN6 cell lines are not commercially available. The cell line was originally published by Miyazaki, J.-I. et al. Establishment of a Pancreatic β Cell Line That Retains Glucose-Inducible Insulin Secretion: Special Reference to Expression of Glucose Transporter Isoforms*. Endocrinology 127, 126-132 (1990). https://doi.org:10.1210/endo-127-1-126. We obtained this cell line from the authors of this manuscript.
Authentication	The authors performed RNA-seq on these cells to verify they express the canonical beta cell transcriptome and lacked non-beta cell expression profiles. The cells were also tested for their ability to secrete insulin in response to glucose stimulation.
Mycoplasma contamination	The cells tested negative for mycoplasma contamination
Commonly misidentified lines (See ICLAC register)	N/A

Animals and other research organisms

Policy information about studies involving animals; ARRIVE guidelines recommended for reporting animal research, and Sex and Gender in Research

Laboratory animals	Mice; Pdx1:CRE (B6.FVB-Tg(Pdx1-cre)6Tuv) and Rbfox2flox (B6.129S2-Rbfox2tm1.1Dblk/J); 8-week-old adult male and female mice.
Wild animals	N/A
Reporting on sex	P0 Weight (Figure S3A) - sex cannot be determined at this early stage<br>P0 Ad lib Blood Glucose (Figure S3B) - sex cannot be determined at this early stage<br>4wk Weight (Figure S3C) 20 males and 13 females<br>4wk Fasting Blood Glucose (Figure S3D) 20 males and 13 females<br>8wk Weight (Figure S3E) 30 males and 17 females<br>8wk Fasting Blood Glucose (figure S3F) 30 males and 27 females<br>4wk Male GTT (Figure S3G-H) 18 males<br>4wk Female GTT (Figure S3I-J) 12 females<br>8wk Female GTT (Figure S3K-L) 7 females<br>16wk Combined GTT (Figure S3M-N) 4 males and 7 females

Rbfox2-mut RNA-Seq (several figures) 4 males and 2 females
Electron Microscopy (Figure 4A-C, S5A-D) 4 males and 2 females
GSIS Males (Figure S6E) 6 males
GSIS Females (Figure S6E) 6 males
Insulin Staining (Figure 2C) 6 males
C peptide Staining(Figure 2D) 6 males
8wk Male GTT (Figure 2F-G) 13 males
8wk Male O-GTT (Figure 2H-I) 11 males
dGSIS (Figure 4D-G) 8 males
Plamsa Insulin (Figure 4H, S5G) 16 males
Insulin Exocytosis (Figure 4I) 8 males
Capacitance/Calcium Current (Figure S5H-I) 8 males

Field-collected samples

N/A

Ethics oversight

The mice were maintained under the University of Colorado Institutional Animal Care and Use Committee (IACUC) approved protocol #00045. Mice were housed by sex with up to 5 littermates per cage in a 22°C room with 12-hour light/dark cycle and unlimited access to food (Inotivco 2920X) and water. Cages were cleaned every two weeks and mice were regularly monitored for injury and infection. Euthanasia was performed with CO2 and cervical dislocation.

Note that full information on the approval of the study protocol must also be provided in the manuscript.
